# Correction: Moumin et al. Usual Nutrient Intake Distribution and Prevalence of Inadequacy among Australian Children 0–24 Months: Findings from the Australian Feeding Infants and Toddlers Study (OzFITS) 2021. *Nutrients* 2022, *14*, 1381

**DOI:** 10.3390/nu15051144

**Published:** 2023-02-24

**Authors:** Najma A. Moumin, Merryn J. Netting, Rebecca K. Golley, Chelsea E. Mauch, Maria Makrides, Tim J. Green

**Affiliations:** 1 Discipline of Pediatrics, Faculty of Health and Medical Sciences, University of Adelaide, Adelaide, SA 5000, Australia; 2 Women and Kids Theme, South Australian Health and Medical Research Institute, Adelaide, SA 5000, Australia; 3 Nutrition Department, Women’s and Children’s Health Network, Adelaide, SA 5006, Australia; 4 Caring Futures Institute, College of Nursing and Health Sciences, Flinders University, Adelaide, SA 5000, Australia

The authors would like to correct an error in their recently published paper [1]. The prevalence of inadequacy for iron for older infants (6–12 months) should be 75%, not 92%, as reported in the paper. The software [2] used to calculate the prevalence of inadequacy requires the user to enter the percentage bioavailability of iron. The Estimated Average Requirement (EAR) for iron in older infants is 7 mg/d and is based on 10% iron bioavailability [3]. When we entered 10% bioavailability, the software set the EAR at 12.6 mg/d, not 7 mg/d; thus, the prevalence of inadequacy was overestimated.

The authors apologize for any inconvenience caused to the readers by the changes. Although the prevalence of inadequacy for iron for older infants has fallen, 75% is still high.

1. Abstract

“Exceptions were iron and zinc where the prevalence of inadequacy was estimated to be 90% and 20%, respectively, for infants aged 6–11.9 months.”

was changed to:

“Exceptions were iron and zinc where the prevalence of inadequacy was estimated to be 75% and 20%, respectively, for infants aged 6–11.9 months.”

2. The following content in the Results section, paragraph 3

“More than 90% of older infants and one quarter of toddlers had inadequate iron intakes (Tables 3 and 4).”

was changed to:

“More than 75% of older infants and one quarter of toddlers had inadequate iron intakes (Tables 3 and 4).”

3. Table 3 in the “Iron^6^, mg/d” row under the “<EAR” column, the number has been changed from 92 to 75.

4. The following content in the Discussion section, paragraph 1

“Exceptions were a very high prevalence of inadequacy for iron (>90%) in infants 6–11.9 months and excessive sodium consumption in a high proportion of toddlers 12–24 m (30%).”

was changed to:

“Exceptions were a very high prevalence of inadequacy for iron (75%) in infants 6–11.9 months and excessive sodium consumption in a high proportion of toddlers 12–24 m (30%).”

5. The following content in the Discussion section, paragraph 3

“For the two nutrients with EARs, iron and zinc, the prevalence of inadequacy was estimated to be 90% and 20%, respectively.”

was changed to:

“For the two nutrients with EARs, iron and zinc, the prevalence of inadequacy was estimated to be 75% and 20%, respectively.”
